# Focal acral hyperkeratosis with response to acitretin

**DOI:** 10.1016/j.jdcr.2024.04.037

**Published:** 2024-05-07

**Authors:** Meghan R. Mansour, Daniel Wenzel, Lori Lowe, Severine Cao

**Affiliations:** a Department of Dermatology, MetroHealth Case Western University Hospital, Cleveland, Ohio; b Department of Dermatology, University of Michigan, Ann Arbor, Michigan; c Department of Pathology, University of Michigan, Ann Arbor, Michigan

**Keywords:** disseminated punctate keratoderma, palmoplantar keratodermas

## Introduction

Focal acral hyperkeratosis (FAH) manifests clinically as multiple crateriform keratotic papules on the palmoplantar margins.^1^ Often, these papules coalesce into plaques, intensifying the roughness of the skin. FAH mirrors acrokeratoelastoidosis and other marginal papular acrokeratodermas (MPAs) clinically but is distinguished by an absence of elastorrhexis on histology.^1^^,^^2^ The majority of reported cases have presented in childhood with an autosomal dominant inheritance pattern, though sporadic forms have also been reported. Documented treatment options have shown limited success.^3^^,^^4^ Amid the spectrum of MPAs, FAH has been sparsely described over the past 15 years,^5^ with its initial description dating back to 1983.^1^ We present a unique case of FAH distinguished by its extensive distribution and significant hyperpigmentation, with a moderate response to acitretin.

## Case presentation

**Fig 1:**
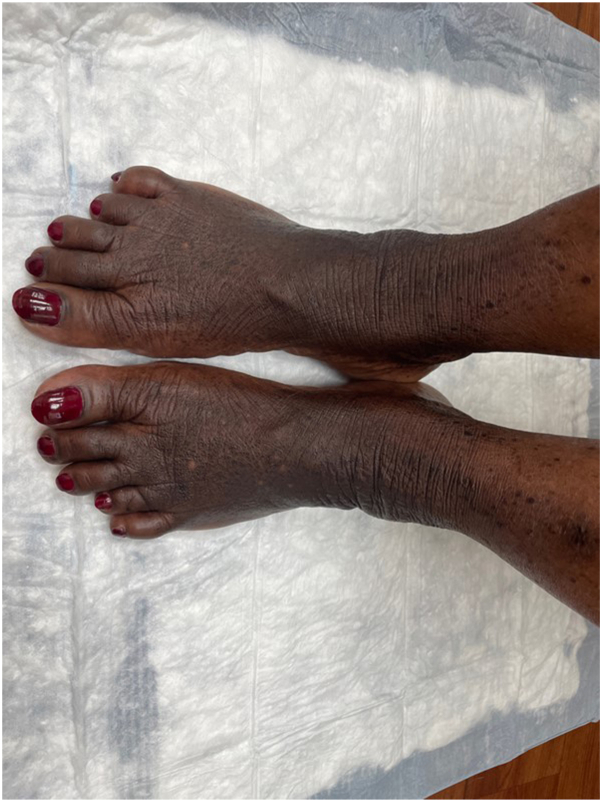
Hyperpigmented plaques *dotted* with punctate depressions on the ankles and dorsal feet, alongside scattered *black* to *dark brown* papules with keratotic rims on the lower legs of a patient with Fitzpatrick skin type 5 with focal acral hyperkeratosis.

**Fig 2:**
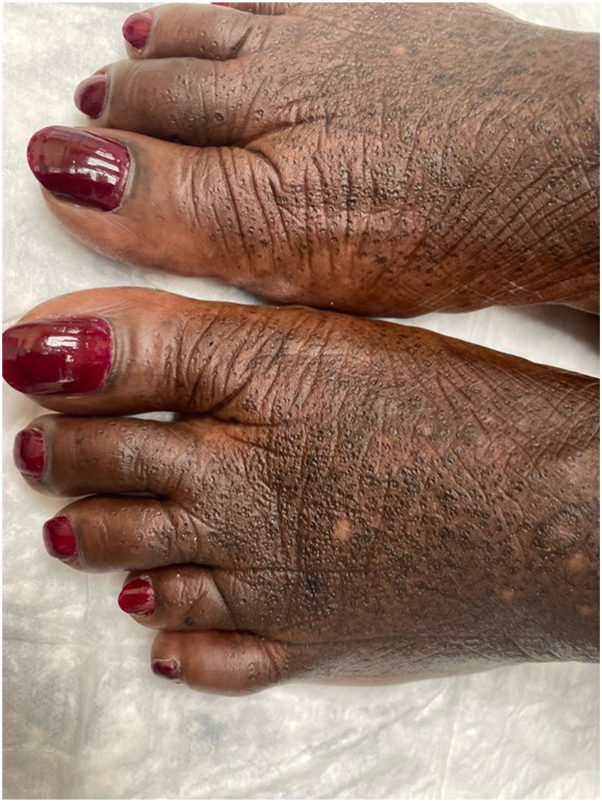
Hyperpigmented plaques *dotted* with punctate depressions on the ankles and dorsal feet.

**Fig 3:**
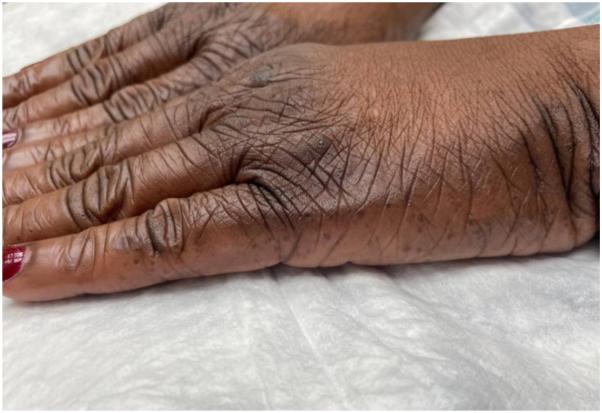
The palmar margins of a patient with Fitzpatrick skin type 5 with hyperpigmented plaques *dotted* with punctate depressions.

**Fig 4:**
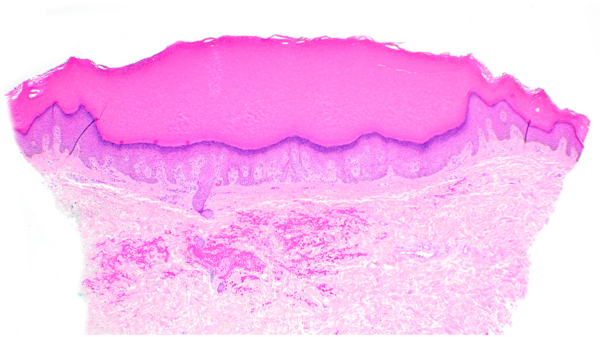
Skin biopsy demonstrated compact orthohyperkeratosis overlying a well-circumscribed epidermal depression (40×, hematoxylin-eosin stain).

A 57-year-old female with Fitzpatrick skin type 5 sought evaluation for skin changes on her hands and feet over the last 10 years. The symptoms started as areas of hyperpigmentation on her feet with gradual formation of black keratotic papules and roughened skin, which later appeared on her lower legs, hands, and wrists. She described associated itching, unrelieved by emollients. Physical examination revealed hyperpigmented plaques dotted with punctate depressions on the ankles and dorsal feet, alongside scattered black to dark brown papules with keratotic rims on the lower legs (Figs 1 and 2). Similar, less prominent changes were observed on the palmar margins (Fig 3) and ventral wrists, with a few punctate depressions on the plantar hands within the dermatoglyphs. The skin felt firm and rough. The patient reported no family members with similar skin findings. She denied recent medication changes. A lesional punch biopsy from the left shin demonstrated a distinct area of compact orthohyperkeratosis overlying an epidermal depression, most consistent with punctate keratoderma (Fig 4). Elastin stains were negative for elastorrhexis. Given the clinical and histological findings, a diagnosis of FAH was favored.

Six months after the patient’s initial dermatology visit, she was diagnosed with essential thrombocytosis with JAK2 V617F mutation positivity. The diagnosis came many years after the onset of her skin changes. At the time of publication, her platelet counts were rising and she began aspirin and hydroxyurea for treatment. The patient was otherwise up-to-date with age-appropriate malignancy screening. Arsenic punctate keratoderma was excluded with a normal arsenic level.

Treatment consisted of: urea 40% cream, consistent moisturization, saltwater soaks, and hydroquinone 4% cream. At a 3-month follow-up, she reported the hydroquinone was helpful for lightening her skin, but the roughness and punctate lesions had worsened. Acitretin 25 mg/d was initiated. At a follow-up 6 months after initiation, there was improvement with softening of the skin which was greatly appreciated by the patient. She continues to follow in our clinic.

## Discussion

Punctate palmoplantar keratodermas represent a group of hereditary and acquired disorders characterized by thickened skin on the palms and soles in the form of small keratotic papules or pits, with occasional involvement of dorsal surfaces.^6^ Classification of disease within this category is challenging with many overlapping terms and features. MPAs have emerged as a group within punctate punctate palmoplantar keratoderma presenting with flesh to yellow-colored papules and plaques primarily distributed along the borders of the palms and soles.^2^ Within MPAs, further subtypes have been described, including acrokeratoelastoidosis and FAH, which are similar clinically but distinguished by histologic findings of elastorrhexis seen with acrokeratoelastoidosis, but not FAH.^2^^,^^5^

We present a case of FAH that is unusual due to the wider distribution of lesions, involving not only the margins of the palms and soles but predominantly the dorsal feet and ankles, with scattered involvement on the lower legs, hands, and wrists. Additionally, our case demonstrates presentation in Fitzpatrick skin type 5/6 and highlights the significant associated hyperpigmentation which was of great concern to the patient and must be considered during management.

The clinical differential diagnosis in our case included acrokeratoelastoidosis, porokeratosis palmaris et plantaris et disseminata, and annular lichen planus. The absence of elastorrhexis, cornoid lamellae, and lichenoid interface dermatitis, respectively, reasonably excludes these conditions.

The first initial reports of FAH were observed in patients of African or Middle Eastern origin.^1^ More recent cases of FAH have been documented in people of Caucasian and Asian background, negating any racial or ethnic predominance.^5^ A subset of cases appears to be familial with a suggestion of autosomal dominant inheritance, though acquired forms have also been described.^1^ Current literature suggests that acquired keratodermas are primarily seen in male patients. However, when they arise in female patients, they present after the second decade of life and/or following menopause,^6^^,^^7^ consistent with our case findings.

Although acquired keratodermas have been linked to malignancies, especially gastrointestinal and pulmonary tumors^8^; the specific subcategory of MPAs has not been reported to be associated with malignancy. Our patient was noted to have essential thrombocytosis undergoing active treatment. The onset of her malignancy was 10 years after the onset of her skin disease, but a possible association cannot be ruled out. For individuals with acquired FAH, adherence to age-appropriate cancer screening and consistent follow-up could be considered.^8^

Treatment of FAH is difficult and aimed at reducing the roughness of the skin. Several therapies have been tried, including liquid nitrogen cryotherapy, keratolytics like urea and salicylic acid, topical and systemic retinoids, and topical and systemic steroids, all with limited results.^9^ Of note, 1 case reported sustained improvement after use of topical calcipotriol twice daily for 8 weeks, with no recurrence at follow-up in 8 months.^9^ As shown in our case, FAH can present with significant hyperpigmentation, which can be treated with topical lightening creams such as hydroquinone. Unfortunately, our patient was refractory to topical keratolytics. She was started on acitretin and at 6 months follow-up, reported improvement in the roughness of her skin. Our case represents the first to report a response to acitretin.^5^

In conclusion, we present a unique case of FAH with widespread distribution and significant hyperpigmentation. We also suggest a potential association with malignancy and demonstrate a response to acitretin. We hope our case aids in our recognition and understanding of this rare and still poorly understood disease.

## Conflicts of interest

None disclosed.
